# Efficient Decoupled Electrolytic Water Splitting in Acid through Pseudocapacitive TiO_2_

**DOI:** 10.1002/advs.202401261

**Published:** 2024-05-14

**Authors:** Mairis Iesalnieks, Mārtiņš Vanags, Linda Laima Alsiņa, Raivis Eglītis, Līga Grīnberga, Peter C. Sherrell, Andris Šutka

**Affiliations:** ^1^ Institute of Materials and Surface Engineering Faculty of Natural Sciences and Technology Riga Technical University P. Valdena Street 3/7 Riga LV‐1048 Latvia; ^2^ Institute of Solid State Physics University of Latvia Riga LV‐1063 Latvia; ^3^ Applied Chemistry & Environmental Science School of Science RMIT University 124 La Trobe St Melbourne 3000 Australia

**Keywords:** electrolysis, pseudocapacitors, titanium dioxide

## Abstract

Water electrolysis remains a key component in the societal transition to green energy. Membrane electrolyzers are the state‐of‐the‐art technology for water electrolysis, relying on 80 °C operation in highly alkaline electrolytes, which is undesirable for many of the myriad end‐use cases for electrolytic water splitting. Herein, an alternative water electrolysis process, decoupled electrolysis, is described which performed in mild acidic conditions with excellent efficiencies. Decoupled electrolysis sequentially performs the oxygen evolution reaction (OER) and the hydrogen evolution reaction (HER), at the same catalyst. Here, H^+^ ions generated from the OER are stored through pseudocapacitive (redox) charge storage, and released to drive the HER. Here, decoupled electrolysis is demonstrated using cheap, abundant, TiO_2_ for the first time. To achieve decoupled acid electrolysis, ultra‐small anatase TiO_2_ particles (4.5 nm diameter) are prepared. These ultra‐small TiO_2_ particles supported on a carbon felt electrode show a highly electrochemical surface area with a capacitance of 375 F g^−1^. When these electrodes are tested for decoupled water splitting an overall energy efficiency of 52.4% is observed, with excellent stability over 3000 cycles of testing. This technology can provide a viable alternative to membrane electrolyzers—eliminating the need for highly alkaline electrolytes and elevated temperatures.

## Graphical abstract

A new path for water electrolysis is described, decoupled water electrolysis, by exploiting cheap, abundant, pseudocapacitive TiO_2_ nanoparticles in acidic conditions. This two‐step water‐splitting reaction is stable over 3000 cycles and demonstrates an overall 52.4% efficiency in acid at room temperature. This concept opens a path for water‐splitting technologies, from electrolyzer design to hydrogen storage.

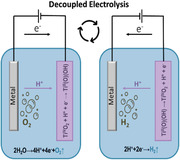

## 1 Introduction

Hydrogen is considered a versatile and clean energy carrier as it can be produced by water electrolysis, electrochemically splitting water molecules into hydrogen and oxygen.^[^ ^1^ ^]^ Green hydrogen is produced when electrolysis is performed using only renewable energy such as solar or wind power^[^ ^2^ ^]^ and can help with grid equalizing in place of batteries.^[^ ^3^ ^]^ The produced hydrogen can then be stored prior to use as a clean fuel, reacting with oxygen to produce water, with zero greenhouse gas emissions, being touted for many applications, including fuel cells in transport,^[^ ^4^ ^]^ for blending with natural gas for combustion,^[^ ^5^ ^]^ and for chemical production via the Fischer–Tropsch process.^[^ ^6^ ^]^

Currently, commercial water electrolysis is performed predominantly in membrane electrolyzers. However, these membrane electrolyzers have several drawbacks, including high material and catalyst cost, high assembly cost due to device complexity, high maintenance costs, low tolerance to impurities in the electrolyte,^[^ ^7^, ^8^ ^]^ and adverse operating conditions (high temperature, highly alkaline electrolytes), which in turn lead to membrane fouling.^[^ ^9^ ^]^ The membrane electrolysis concept also raises operational safety issues due to H_2_/O_2_ cross‐over across the membrane/separator.

Recently, the decoupled water electrolysis concept has emerged as an alternative to membrane electrolysis. In decoupled electrolysis the oxygen evolution reaction (OER) and hydrogen evolution reaction (HER) are performed sequentially on the same catalyst, thus avoiding the use of membrane, enabling non‐alkaline electrolytes, a wide window of operating conditions, and lowers the requirements related to auxiliary devices for maintenance.^[^ ^10^ ^]^ These advantages make decoupled water electrolysis a promising alternative to explore for achieving green hydrogen production.

In decoupled electrolysis, two main strategies have been explored, by harnessing electron‐coupled proton buffers^[^ ^11^, ^12^, ^13^, ^14^ ^]^; or by utilizing pseudocapacitive electrodes to accumulate OH^−^ or H^+^ ions while they are not being consumed in the catalytic reactions.^[^ ^9^, ^15^, ^16^ ^]^ In pseudocapacitive systems, the OH^−^ or H^+^ ions are stored while the OER is occurring, and released while HER is occurring.

Literature reports of these systems have focused on two main classes of materials, Ni(OH)_2_‐based pseudocapacitors have been exploited for alkaline electrolytes, while WO_3_‐based pseudocapacitors have been exploited for acid‐decoupled electrolysis.^[^ ^17^, ^18^, ^19^ ^]^

In the case of Ni(OH)_2_, in step (1), the H_2_ is released following reaction 4H_2_O + 4e^− ^→ 4OH^− ^+ 2H_2_, with OH^−^ intercalating into the Ni(OH)_2,_ which in turn is transformed to NiOOH by the reaction Ni(OH)_2_ + OH^−^ → NiOOH + H_2_O + e^−9^. The NiOOH is transformed back to Ni(OH)_2_ in step (2)_,_ and oxygen is released. In the case of WO_3_, oxygen is released in step (1) alongside H^+^ intercalation into WO_3_ via 2H_2_O → 4H^+^ + 4e^−^ + O_2_ and xH^+^ + xe^−^ + WO_3_ → H_x_WO_3_, respectively. In step (2), H^+^ is released from the WO_3_ pseudocapacitive electrode and H_2_ is released on the working electrode by the reactions H_x_WO_3_ → xH^+^ + xe^−^ + WO_3_ and 2H^+^ + 2e^−^ → H_2_, respectively. However, while these approaches are promising, both Ni and W present challenges moving forward, with Ni(OH)_2_ exhibiting stability issues^[^ ^15^ ^]^ along with environmental and health risks, and W being considered by the European Union as a critical metal with a high supply risk.^[^ ^20^ ^]^ Recently, carbonaceous materials have been reported for such decoupled electrochemical systems, either directly for water splitting,^[^ ^7^ ^]^ or using the same fundamental concept as a hydrogen battery.^[^ ^21^ ^]^ These recent works highlight the opportunity advantages in the use of cheap, abundant, electrode materials for decoupled electrolysis.

TiO_2_ is one of the most abundant oxides globally and is an excellent pseudocapacitive material that has not been studied for decoupled electrolysis. While small TiO_2_ particles have demonstrated capability for Li^+ [^ ^22^, ^23^ ^]^ and Na^+^ intercalation,^[^ ^24^ ^]^ H^+^ intercalation into TiO_2_ required for decoupled electrolysis is scarcely reported.^[^ ^25^ ^]^ Kim et al.^[^ ^26^ ^]^ demonstrated H^+^ intercalation in high surface area mesoporous amorphous TiO_2_ electrode films via a proton‐coupled electron transfer reaction, equivalent to the Li^+^‐coupled electron transfer reaction.^[^ ^27^ ^]^ In other work, TiO_2_ nanotube layers showed H^+^ intercalation/de‐intercalation ability during cathodic/anodic cycling with a high storage capacity.^[^ ^28^ ^]^ These preliminary suggest the ability of TiO_2_ to support decoupled water electrolysis.

Herein, we prepare ultra‐small (<5 nm diameter) TiO_2_ nanoparticles in a conductive carbon black matrix and probe their performance in decoupled water electrolysis.^[^ ^29^ ^]^ The ultra‐small nanoparticles demonstrate excellent stability over 3000 cycles and an overall electrolysis efficiency of 52.4%, thus opening new opportunities for developing ecofriendly hydrogen generation by exploiting abundant materials.

## 2 Results and Discussion

### 2.1 Concept of Decoupled Water Electrolysis in Acidic Media

**Figure 1:**
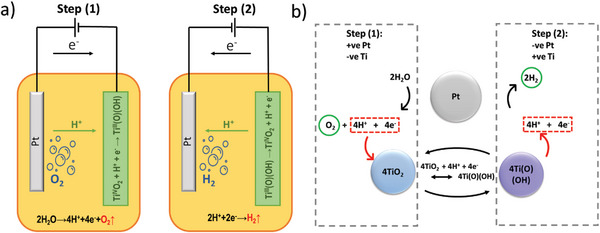
Concept of decoupled water splitting using a TiO_2_ auxiliary electrode; a) visual representation of the electrochemical cell setup; and b) reaction diagram showing cyclical HER and OER reactions.

The decoupled electrolysis principle using pseudocapacitive TiO_2_ in an acidic 0.5 m H_2_SO_4_ solution is demonstrated in **Figure** **1**a. A platinum electrode was used as a model system to cyclically catalyze the 1) OER or 2) HER. To control which reaction occurs on the Pt catalyst, and how ions and charge are stored at the TiO_2_ auxiliary electrode, the polarity of the applied voltage was changed stepwise. In the first water‐splitting cycle, step (1), TiO_2_ is protonated to titanium oxyhydroxide (TiO_2_ + e^−^ + H^+^ → Ti(O)(OH)) in acidic media^[^ ^30^ ^]^ following the Pourbaix diagram.^[^ ^31^ ^]^ Step (1) is energetically costly as the OER (2H_2_O → O_2_ + 4e^−^ + 4H^+^), requiring four electrons to proceed. When the cell polarity is reversed, in step (2), the H^+^ ions stored at the TiO_2_ electrode in step (1) are released to enable the HER on the Pt catalyst. Step (2) then continues until Ti(O)(OH) is fully converted back to TiO_2_, where the cycle is repeated (Figure 1b). As gases are generated from ions released from the TiO_2_ electrode, and HER occurs in acidic media, hydrogen generation at the second step is theoretically highly energy efficient/beneficial, as described below.

### 2.2 TiO_2_ Synthesis and Characterization

**Figure 2:**
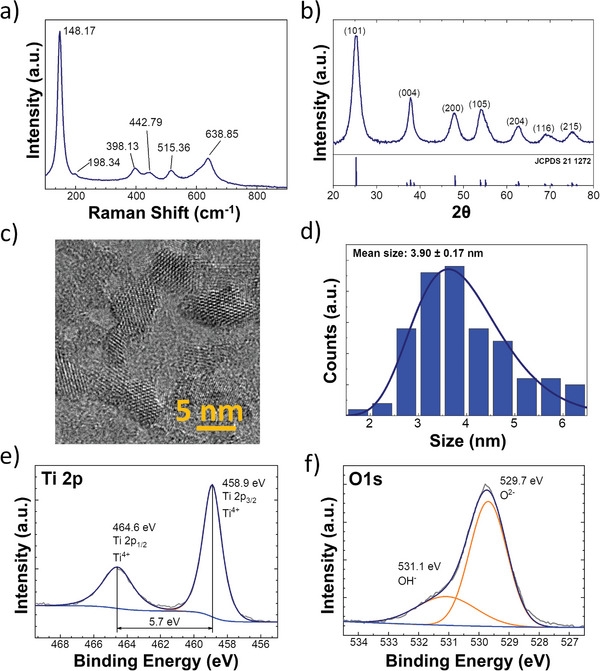
Characterization of synthesized TiO_2_ nanoparticles after surfactant stripping; a) Raman spectroscopy; b) XRD diffractogram with JCPDS 21–1272 standard; c) TEM image of synthesized particles; d) Histogram of particle size distribution obtained from analysis of 120 particles via TEM; and high‐resolution XPS spectra for; e) titanium 2p; and f) oxygen 1s.

To achieve the concept described above, high surface area TiO_2_ is required. TiO_2_ was produced sol–gel synthesis (see Experimental Section for full details), producing ultra‐small anatase nanoparticles. The anatase structure was confirmed by Raman spectroscopy (**Figure** **2**a) and X‐ray diffraction (XRD) (JCPDS 21–1272) (Figure 2b). The Raman spectra in Figure 2a show peaks at 148.17 cm^−1^ (*E*_g_), 198.34 cm^−1^ (*E*_g_), 398.13 cm^−1^ (*B* ^1^_g_), 515.36 cm^−1^ (*A* ^1^_g_), and 638.85 cm^−1^ (*E*_g_) corresponding to TiO_2_ anatase vibrational modes.^[^ ^32^ ^]^ A slight Raman band shift can be observed in Raman spectroscopy due to the small size of synthesized nanoparticles compared to the literature.^[^ ^33^ ^]^ The crystallites size was calculated to be 4.54 nm from the average of all XRD peaks, via the Scherrers’ equation:^[^ ^34^ ^]^

(1) $$D=\frac{k\lambda }{B\cos\theta }$$

where *D* is the crystallite size (nm); k is the particle form factor (0.9); λ is the wavelength of the X‐ray source (0.154056 nm); B is the peak full width at half maximum height; and θ is the Braggs angle.

Transmission electron microscopy (TEM) analysis (Figure 2c) shows individual TiO_2_ nanoparticles, with an average size of 3.90 ± 0.17 nm, aligned with the calculated size from XRD.

X‐ray photoelectron spectroscopy (XPS) of the Ti 2p and O 1s regions are shown in Figure 2e,f respectively. Binding energies of the Ti 2p_3/2_ peak at 458.9 EV indicate the presence of a Ti^4+^ oxidation state without a lower oxidation state‐ Ti^3+^. Peak splitting between Ti 2p3/2 and Ti 2p1/2 was determined to be 5.7 eV, indicating the presence of anatase.^[^ ^35^ ^]^ Oxide lattice oxygen formed the majority of O 1s spectra, with ≈26 (±1%) of ─OH surface hydroxyl component at a binding energy of 531.1 eV, corresponding to the first couple of monolayers of surface hydroxyl.^[^ ^36^ ^]^

### 2.3 Electrochemical Properties of Auxiliary TiO_2_ Electrodes

To identify the suitability of the anatase TiO_2_ particles for the acidic, decoupled electrochemical water splitting reaction cyclic voltammetry (CV) was performed.

**Figure 3:**
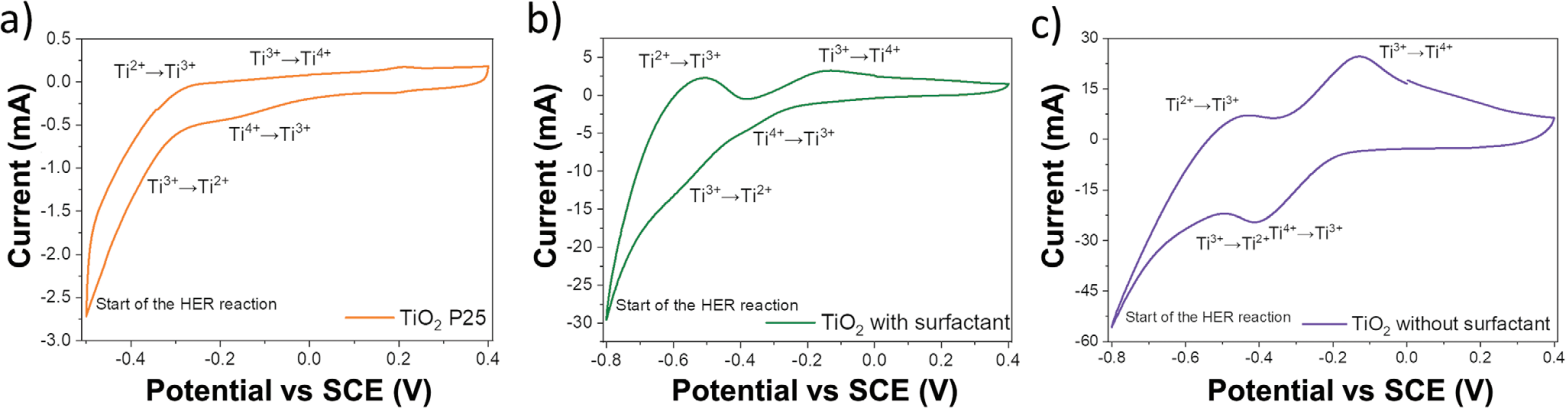
Cyclic voltammetry (CV) curves for a) TiO_2_ P25, b) TiO_2_ with surfactant, and c) TiO_2_ without surfactant nanoparticle electrodes. Curves were taken in 0.5 m H_2_SO_4_ electrolyte in a three‐electrode cell with a scan rate of 5 mV s^−1^.

In **Figure** **3**a, the CV of a commercial “Aeroxide P25” TiO_2_ electrode is shown (Figure S3, Supporting Information), these particles have a diameter of ≈25 nm. Between an applied voltage of −0.3 and 0.4 V versus the saturated calomel electrode (SCE), only electrochemical double‐layer capacitance is observed. However, as the voltage drops below −0.3 V versus SCE, the cathodic current increases rapidly representing electrolyte breakdown, in this case, the HER. Hydrogen gas bubbles were visually observed to be released below this voltage, so the voltage in the cycle was limited to −0.5 V.

In comparison, the CV curves of the as synthesized ultra‐small TiO_2_ nanoparticles (Figure 3b,c) differ markedly from the CV curve of the TiO_2_ P25 sample. Here, two configurations of ultra‐small TiO_2_ nanoparticle electrodes were studied; as produced (Figure 3b), and after stripping of the surfactant using hexane to expose the pristine TiO_2_ surface (Figure 3c). The as‐produced particles remain partially coated in the surfactant used for synthesis, 4‐dodecylbenzene sulphonic acid (4‐DDBSA). For the as‐produced TiO_2_ nanoparticles (with surfactant), as the potential decreased below −0.25 V versus SCE, a pronounced cathodic current appears, which forms an indistinct reduction peak at ≈−0.38 V versus SCE. A secondary reduction peak is observed −0.58 V versus SCE, with hydrogen gas evolution being observed at potentials below −0.7 V versus SCE. As the potential is increased, an oxidation peak appears at −0.5 V, followed by another oxidation peak at −0.13 V, followed by electrochemical double layer formation.

In the CV curve of the stripped surfactant‐free TiO_2_ NPs (Figure 3c), the reduction and oxidation peaks clearly appear at −0.4 V versus SCE and −0.13 V versus SCE, respectively. This redox process corresponds to the following reaction:^[^ ^37^ ^]^

(2) $$\mathrm{Ti}O_{2}+e^{-}+H_{3}O^{+}↔\mathrm{Ti}\left(O\right)\mathrm{OH}+H_{2}O$$

To understand the different CV shapes for P25, and ultra‐small TiO_2_ particles with and without surfactant, the H^+^ intercalation process must be considered. H^+^ intercalation can only occur at the surface of TiO_2_ particles,^[^ ^38^ ^]^ and therefore the surface area:volume ratio of these systems is a key parameter. This surface area:volume ratio inversely scales with particle size, and thus, in the case of the large P25 TiO_2_ particles minimal H^+^ intercalation can occur. In contrast, the ultra‐small TiO_2_ particles have a dramatically increased surface area to volume ratio, enabling significant H^+^ intercalation to occur for the same mass of particles. Further, the stripping of surfactant exposes the full surface area of the ultra‐small TiO_2_ samples to the electrolyte, leading to optimize H^+^ intercalation and thus a maximum for the observed redox peaks. Thus, the stripped ultra‐small TiO_2_ nanoparticles exhibit the strongest redox features, showing that the reaction (1) dominates in the particles (Figure 3c).

**Figure 4:**
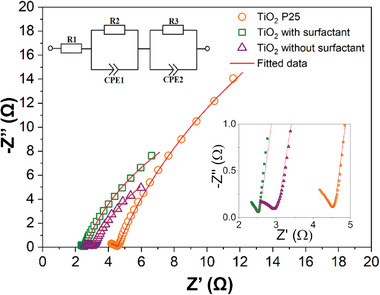
Nyquist plots in complex plane for all three TiO_2_ samples. (Inset: fitted equivalent circuit model).

Electrochemical impedance spectroscopy (EIS) studies were performed at open circuit potential in the same cell used in CV measurements to understand the electrolyte–electrode interface (**Figure** **4**). Two incomplete semicircles characterize plots for all three samples. A semicircle of a small diameter appears in the high‐frequency region, while a semicircle of a large diameter appears in the mid and low‐frequency region. For each Nyquist plot, the equivalent circuit was simulated (Figure 4 inset).

The circuit consists of two parallel circuits of resistance (R) and constant phase elements (CPEs), namely R2, CPE1, and R3 CPE2. The scheme also has a series resistance R1, which characterizes the resistance of the wires, the conductive substrate and the electrolyte.^[^ ^39^ ^]^ The first parallel connection, consisting of CPE1 and R2, characterizes the solvent molecules' geometric capacitance and orientational polarization resistance. This connection purely characterizes the surface boundary layer between electrode and electrolyte with no Faradaic response. The second parallel connection, R3 and CPE2, characterizes the double‐layer capacitance on the boundary surface of the electrode‐electrolyte and the charge transfer resistance.^[^ ^40^ ^]^ This charge transfer resistance is related to the electron transfer resistance of the reaction (2). By analyzing the values of the simulated parameters summarized in **Table** **1**, we can observe that the value of R1 is approximately similar for all samples since the wires, base, and electrolyte were the same in all three cases. The R2 values are also similar in the border of error for all three samples since the solvent was water in all cases.

**Table 1:** Equivalent circuit parameter values from Nyquist plots.

Parameter		TiO_2_ P25		TiO_2_ with surfactant		TiO_2_ without surfactant	
		Value	Error [%]	Value	Error [%]	Value	Error [%]
R1	Ω	3.1	±0.5	0.5	±1.5	2.0	±0.7
R2	Ω	1.5	±3.1	2.1	±3.4	1.1	±1.1
CPE1	Y1, Ω∙s^N1^	0.327	±7.4	0.946	±9.3	9.56	±8.7
	N1	0.58	±6.2	0.35	±7.4	0.40	±2.3
R3	Ω	82.7	±6.5	35.8	±7.2	27.4	±5.7
CPE2	Y2, Ω∙s^N2^	26.7	±1.2	26.2	±2.1	229	±0.6
	N2	0.82	±0.6	0.83	±0.8	0.80	±0.5

Noticeable differences between the samples appear in the CPE1 parameters Y1 and N1. According to the obtained results, the Y1 value is 0.327 for the TiO_2_ P25 sample, 0.946 for the ultra‐small TiO_2_ and 9.56 for the stripped ultra‐small TiO_2_. Y1 almost inherits capacitance, but if in an ideal capacitor, the phase shift is $\frac{\pi }{2}$, then the phase shift of the CPE is $N\cdot \frac{\pi }{2}$, where N varies from 0 to 1.^[^ ^7^ ^]^ Therefore, Y values will be used for capacity characterization in the following because the N values are sufficiently similar to the corresponding CPE. The small capacity of the TiO_2_ P25 sample can be explained by the much larger particle size (25 nm) compared to the ultra‐small TiO_2_ particles (≈4 nm). Therefore, the surface area in contact with the electrolyte for such small particles is much larger, and the surface capacitance also increases. There is also a significant difference in the surface characteristic capacitance parameter between the synthesized ultra‐small TiO_2_ before and after surfactant stripping. This difference arises as the surfactant prevents the electrolyte from interacting with the active TiO_2_ surface, leading to decreased capacitance and the electrode | electrolyte interface. The R3 parameter in Table 1 is the smallest for synthesized ultra‐small TiO_2_ stripped from surfactant due to the lower charge transfer resistance. The ion intercalation also occurs on the surface of large‐size TiO_2_ nanoparticles. The charge transfer resistance for the particles covered by a surfactant is slightly higher due to hindered charge exchange. The Y2 parameter of CPE2, characterizing electric double‐layer capacitance, is ≈229 for stripped TiO_2_ particles and 26 for as‐prepared and P25 TiO_2_ nanoparticles. Again, the benefits of stripped ultra‐fine TiO_2_ particles can be seen because the double‐layer capacitance is directly proportional to the interfacial area between the electrode and the electrolyte. This is also correlated with the CV measurements. The capacitance of the P25 sample is the same as that of the as‐prepared ultra‐small TiO_2_ particles because the surfactant blocks the particle's surface, as discussed previously.

This improved capacitance was studied by CV (Figures S4 and S5, Supporting Information), with the capacitance increasing from 30 F g^−1^ (P25); to 326 F g^−1^ (ultra‐small TiO_2_ with surfactant); and 375 F g^−1^ (ultra‐small TiO_2_ without surfactant)—highlighting the extra available surface attained by increasing surface area.

**Figure 5:**
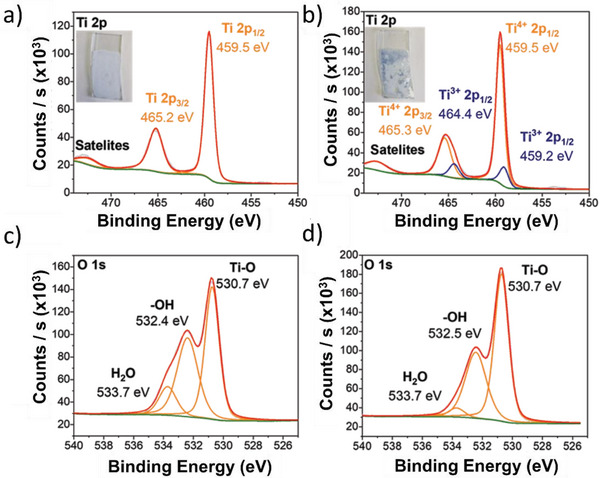
XPS data for TiO_2_ without surfactant on ITO substrates; a,c) as produced and b,d) H^+^ intercalated.

In order to understand and prove the phase changes detected electrochemically, the intercalation process for TiO_2_ was analyzed by using XPS in intercalated and deintercalated states (**Figure** **5**). To detect the presence of Ti (III) and other lower oxides of titanium, pure TiO_2_ suspension in water was deposited onto an ITO substrate and subjected to the same conditions as chronopotentiometry with a charging current of 10 mA. A color change of TiO_2_ coating to blue was observed, indicating changes in material structure. The color starts to change back to its original state of white, as soon as the sample is exposed back to air, and thus the measured XPS also represents a mixture of these blue/white states. This instability explains the low Ti^3+^ concentration (14.8%) in Ti 2p spectra. In the intercalated stage, we can observe the presence of lower oxidation states of titanium. The O 1s spectra reveal the presence of three distinctive maxima at 530.7, 532.4, and 533.7 eV, which corresponds to Ti─O bonding, ─OH groups and the possible presence of adsorbed water and organic pollutants on the sample surface respectively.

### 2.4 Chronopotentiometry

**Figure 6:**
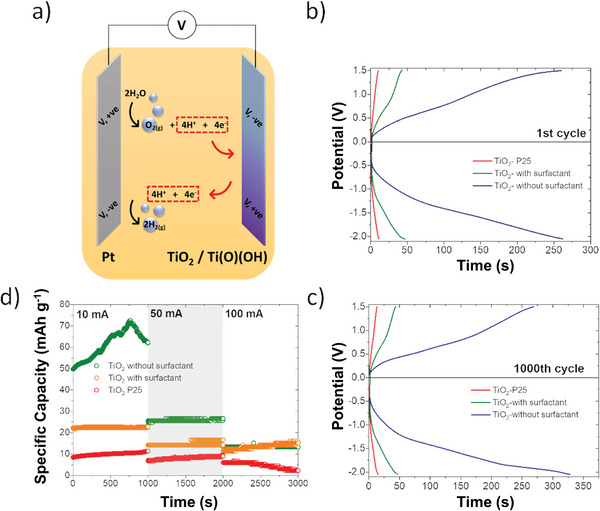
a) Schematic of the processes during chronopotentiometry measurements; b,c) Chronopotentiometry measurements at b) 1st cycle of all three TiO_2_/carbon felt electrode samples, c) the 1000th cycle of all three samples; and d) long‐term durability at different current densities (data shown is the average of three samples for each current density).

With a strong understanding of the electrochemical properties of the ultra‐small TiO_2_ electrodes, decoupled electrolysis was tested under real conditions using chronopotentiometry. Here, a two‐electrode cell was used, where the TiO_2_/carbon felt electrode (with a TiO_2_ loading of 7 mg cm^−2^) was used as the working electrode and the Pt rod was used as the auxiliary electrode. During the chronoamperometry experiments, 10 mA of current was applied to the TiO_2_/carbon felt electrode until a potential of 1.5 V was reached, with the corresponding change in potential over time recorded, with the polarity of the applied current then reversed. Accordingly, OER or HER occurred on the Pt working electrode with intercalated or deintercalated H^+^ ions on the auxiliary electrode (**Figure** **6**a).

Figure 6b shows the first cycles of intercalation and deintercalation during chronopotentiometry. When a negative potential is measured at the TiO_2_/carbon felt electrode, OER occurs at the Pt Working electrode:

(3) $$2H_{2}O\to 4H^{+}+4e^{-}+O_{2}$$

And an intercalation reaction occurs at the negative potential TiO_2_ auxiliary electrode.

(4) $$\mathrm{Ti}O_{2}+xe^{-}+xH^{+}\to H_{x}\mathrm{Ti}O_{2}+xH_{2}O$$

In contrast, when a positive potential is measured at the TiO_2_/carbon felt electrode, a deintercalation reaction subsequently occurs:

(5) $$H_{x}\mathrm{Ti}O_{2}+xH_{2}O\to \mathrm{Ti}O_{2}+xe^{-}+xH^{+}$$

with hydrogen evolution reaction on negative Pt rod:

(6) $$xH^{+}+xe^{-}\to x/2H_{2}$$

From the shape of the chronopotentiometry curves, it can be concluded that the TiO_2_ electrodes behave like supercapacitor electrodes, as no plateau appears.^[^ ^41^ ^]^ The time taken for intercalation and deintercalation to occur, defined by the time to reach an electrode potential of −2.05 V versus OCP and +1.5 V versus OCP respectively, is proportional to the charge storage capacity of the TiO_2_/carbon felt electrode. The differences in the intercalation and deintercalation cycle times indicate the faradic efficiency of the process at 93.4%.

The ultra‐small TiO_2_‐without surfactant sample demonstrates significant storage capacity, with the first intercalation cycle taking 262 s, while the deintercalation took 260 s (Figure 6b). For the sample TiO_2_ with surfactant, the times of these cycles are 47 and 43 s, respectively, and for the sample TiO_2_ P25, 11.2 and 10.5 s (Figure 6b). The intercalation and deintercalation cycle time of 1000th cycles for TiO_2_ without surfactant lasts 328 and 269 s, respectively. For TiO_2_ with surfactant, 47 and 44 s, while for the TiO_2_ P25 sample, 15.2 and 13.3 s (Figure 6c). In situ H_2_ sensors linked to a gas chromatograph were used to prove the selective evolution of H_2_ gas during Step (2) (Figure S7, Supporting Information), supporting the overall mechanism.

Over the total 1000 cycles, both the P25 and TiO_2_‐without surfactant samples showed an increase in cycle duration over cycling (Figure 6d; Figure S6, Supporting Information). A spontaneous deintercalation process can explain this difference at longer cycle times. However, the TiO_2_‐with surfactant sample showed a nearly 100% stability over the 1000 cycles, which is attributed to the surfactant protecting the TiO_2_ particles from changing (via aggregation or disintegration) over the reaction process.

The chronopotentiometry test was also performed at higher currents to achieve a higher throughput of the water‐splitting reaction, approaching commercially relevant current densities namely 50 and 100 mA. At a current of 50 mA, the TiO_2_‐without surfactant sample demonstrates the best performance, however, at a current of 100 mA the charge capacity of the TiO_2_‐with surfactant sample is equivalent. The decrease in the capacity of the TiO_2_ electrodes at higher chronopotentiometry currents is due to the fact that a larger voltage drop occurs through the film and less mass of the active substance participates in the intercalation reaction. This voltage drop is higher for the TiO_2_‐without surfactant compared to TiO_2_‐with surfactant, due to a higher resistance value (Figure 4, Table 1, R1 = 2.0 vs R1 = 0.5), thus bringing these two samples to comparable performance at higher current densities despite the improved surface activity of TiO_2_‐without surfactant. The surfactant here is thought to stabilize the ultra‐small TiO_2_ particles enabling improved rate performance. The TiO_2_ electrodes were studied using SEM (Figure S8, Supporting Information) before and after electrochemical cycling, with no significant changes in electrode morphology being observed.

### 2.5 Efficiency

To assess the efficiency of the decoupled water‐splitting reaction using TiO_2_ electrodes, it is necessary to quantify the amount of hydrogen produced during step (2) of the decoupled water‐splitting reaction. To achieve this, the volumetric displacement method, described in detail in,^[^ ^15^, ^17^ ^]^ was used to determine the flow rate of the released gas. With this method, gas evolution was determined for a sample without surfactant at a current density of 10 mA. The time to produce 0.13 mL of H_2_ gas in twelve measurements is shown in the supplementary information Table S1 (Supporting Information). The faradaic efficiency coefficient was also calculated for each test using the following formula:

(7) $$\eta =\frac{k\times V\times F}{V_{M}\times I\times t}$$

where *V_M_* = 22.4 L mol^−1^ is volumetric Avogadro number, *F* (C mol^−1^) is Faraday constant, *V* (L) is the displaced gas volume per time *t* (s), *I* (A) is current and *k = 2* in HER reaction.

At a current density of 10 mA, the faradaic efficiency average of 12 measurements is 93.4%.

Knowing the faradaic efficiency, the energy efficiency was calculated using the following formula:

(8) $$\eta =\frac{HHV\times M_{mol}\times \eta_{F}\times t}{F\times \int V\left(t\right)dt}$$

*HHV* is the highest heating value for hydrogen gas (140 MJ kg^−1^), *M_mol_* is hydrogen molar mass (0.001 kg mol^−1^), η_*F*_ is faradaic efficiency, *t* (s) is chronopotentiometry cycle time, *F* (C mol^−1^) is faraday constant and integral in the denominator is the area under the voltage curve over time. Only the deintercalation cycle time should be taken in nominator, as hydrogen is produced in this cycle only. If the efficiency of the deintercalation cycle is calculated, then only the integral of the deintercalation cycle is taken, but if total efficiency, then both the intercalation integral and the deintercalation.

Based on the calculations, the total energy efficiency of the overall decoupled water‐splitting reaction is 52.4%, which is comparable to membrane electrolysis (60–80%).^[^ ^45^ ^]^ The efficiency of the deintercalation cycle reaches 167% in relation to the consumed electricity. These calculations correspond to the TiO_2_‐without surfactant sample at 10 mA current (100 mA g^−1^ current density) in chronopotentiometry measurements.

These efficiency values, operating temperature, voltage window and current density are competitive with current electrolyzer systems, summarized in **Table** **2**.^[^ ^42^ ^]^

**Table 2:** Comparison of key operating parameters for current electrolyzer technologies compared to those reported in this work.^[^ ^42^ ^]^

	Alkaline electrolyzer	PEM electrolyzer	Solid oxide electrolyzer	This work
Operating Temperature	60–80 °C	80 °C	600–900 °C	RT
Current Density	Up to 400 mA cm^−2^	Up to 1000 mA cm^−2^	Up to 3200 mA cm^−2^	100 mA cm^−2^
Potential Window	1.8–2.5 V	1.7 V	1.1 V	1.5–2.0 V
Efficiency	Up to 67%	80%	90%	52.4%

As can be seen from the results, such a decoupled electrolysis cell is strongly asymmetric. Namely, the intercalation cycle is extremely disadvantageous, while the deintercalation, during which H_2_ gas is produced, is advantageous. The overall efficiency still remains at the level of efficiency of electrolyzers available in today's industry.^[^ ^43^ ^]^ It is also important to compare the efficiency of decoupled electrolysis with the conventional electrolysis efficiency of the same cell. Namely, by determining the potential of a traditional electrolysis cell at a specific current and dividing this potential by the average potential value of the OER and HER cycle in decoupled electrolysis mode at the same current, it is possible to compare decoupled to traditional electrolysis.^[^ ^44^ ^]^ The average value of the potential in a cell with a platinum cathode and anode during a period of 250 s is 2.16 V (Figure S9, Supporting Information, shows the chronopotentiometry data). The average potential values of HER and OER cycle are 0.94 and 1.51 V for the P25 sample; 0.76 and 1.57 V for the sample with surfactant and 0.92 and 1.49 V for the sample without surfactant. These values were obtained from the Figure 6b. Thus, the comparative energy efficiency coefficients against traditional electrolysis are 88% for the P25 sample, 90% for the sample without surfactant and 93% for the sample with surfactant. Therefore, the energy efficiency of decoupled electrolysis is only ≈10% behind that of traditional electrolysis, maintaining all of the aforementioned advantages.

## 3 Conclusion

Herein we have demonstrated the concept of decoupled electrolysis by exploiting charge storage and H^+^ intercalation at cheap, abundant, pseudocapacitive TiO_2_ electrodes. 4.5 nm TiO_2_ particles deposited on carbon cloth showed an excellent pseudocapacitive response of up to 375 F g^−1^. This high electrochemical surface area (denoted by the high capacitance) enabled extremely effective intercalation and deintercalation of H+ cations on the TiO_2_ during decoupled water splitting. Interestingly, the bare TiO_2_ electrode (without surfactant) showed impressive performance at lower current densities (ca. 100 mA g^−1^), a TiO_2_ which was coated with a surfactant showed equivalent performance and greater stability at higher, commercially relevant current densities (ca. 1 A g^−1^). The overall efficiency of the decoupled water splitting reaction was calculated as 52.4% approaching values for commercial membrane electrolyzers, which operate at 80 °C in highly alkaline conditions. Further improvements in optimizing the efficiency of the oxygen evolution half‐reaction would present dramatic savings in energy opening up this technology for scalable applications.

## 4 Experimental Section

### Synthesis of TiO_2_ Nanoparticles

The TiO_2_ nanoparticle synthesis procedure was based on a modified version of sol–gel synthesis presented by Emmanuel Scolan and Clement Sanchez.^[^ ^45^, ^46^ ^]^ Titanium tetra *n*‐butoxide (9.05 mL, 97%, Sigma–Aldrich) was added dropwise to the mixture of 12.36 mL of *n*‐butanol (≥99.5%, Merch, stored over CaH_2_) and 8.27 mL of acetylacetone (≥99% Merck). Following this, the reaction was brought up to reflux, and a preheated solution of 1.76 g of 4‐dodecylbenzene sulfonic acid (4‐DDBSA, ≥95%, Sigma–Aldrich) in 4.865 g of deionized water was added dropwise to the reaction mixture. After adding the 4‐DBSSA, the solution was left to reflux overnight. The formation of a clear yellow solution could be observed. After cooling the mixture, a particle precipitate was formed, which was then separated and washed with methanol (gradient grade for liquid chromatography, Supelco) using a centrifuge (2‐16P, Sigma) at 2000 × *g* for 1 h; after all washing cycles, particles were left to separate at 4000 × *g* for 1 h and dispersed in *N*, *N*‐dimethylformamide (DMF) (99%, Merck) with fixed concentration at 100 g L^−1^.

For removal of surfactant, 39 mL of hexane (≥97%, Merck) was added to 13 mL of TiO_2_ colloid in DMF. Thirteen milliliters of triethyloxonium tetrafluoroborate (EtO_3_BF_4_, ≥97%, Sigma‐Aldrich) solution in dichloromethane (DCM, for analysis, Supelco) with concentration of 20 mg mL^−1^ was added dropwise, followed by 20 mL of toluene (≥99.7%, Merck). The mixture was mixed for a short period and allowed to separate. After separation, particles were washed with methanol, as stated previously. After washing, particles were dispersed in DMF with a fixed concentration of 100 g L^−1^. Electrochemical properties were evaluated for TiO_2_ nanoparticles with and without surfactant.

### Electrode Preparation

Electrochemical measurements were performed using the ink method. TiO_2_ colloid was mixed with conductive carbon black (Vulcan XC72, Nanografi) and polyvinylidene fluoride (PVDF, average *M*_w_ 530 000, Sigma–Aldrich) in a mass ratio of 6:3:1, respectively. Extra DMF was added to obtain suspension with the desired viscosity. The suspension was stirred for 24 h using a magnetic stirrer and periodical agitation using an ultrasonic bath until a homogenous mixture was achieved. Commercially available carbon felt (AvCarb, resistance 2 Ω cm^−2^, fiber diameter of 20 µm) was used to produce high‐capacity electrodes. Carbon felt was washed using ultrasound for 30 min in a mixture of sulfuric and nitric acids (volume ratio 1:2), followed by 15 min in deionized water, acetone, ethanol, and deionized water. After all washing cycles, carbon felt samples were dried at 60 °C overnight. Electrodes were prepared by impregnating previously washed carbon felt with a prepared ink solution. After immersion, samples were placed on filter paper and dried at 60 °C overnight. Samples using commercially available TiO_2_ (Aeroxide P25, Thermo Fisher) were prepared similarly by dispersing particles in DMF before adding CB and PVDF.

### Sample Characterization

TiO_2_ nanoparticles were characterized using XRD (X'Pert, PANalytical), Raman spectrometry (inVita, Renishaw), and transmission electron spectroscopy (TEM, Tecnai G20, FEI) imaging at 200 kV. Samples in TEM were analyzed by placing them on perforated carbon film on a 400‐mesh copper grid (S147‐4, Agar Scientific). Particle size distribution was determined by analyzing images in ImageJ software. XPS (ESCALAB Xi+, Thermo Scientific) was used to determine oxygen vacancies. Experimental XPS data were analyzed using Avantage 5 software, setting an advantageous carbon peak at 284.8 eV as a calibration point.^[^ ^47^ ^]^ Electrode morphology was evaluated using scanning electron microscopy (SEM, NanoSEM 650, FEI) and energy dispersion spectroscopy EDS (AMETEK AplloX‐SDD).

### Electrochemical Measurements

Electrochemical measurements were performed using a potentiostat (PGSTAT302N, AutoLab). The cyclic voltammetry (CV) was performed using a three‐electrode cell with a high‐capacity TiO_2_ electrode as a working electrode (WE), a 2 mm Pt rod (Metrohm AG) as the reference electrode (RE), and a SCE (Hanna Instruments) as the counter electrode (CE) (See Figure S1, Supporting Information). H_2_SO_4_ (0.5 m) solution was used as an electrolyte. CVs were performed at different scan rates from 2 to 100 mV s^−1^. CV curves were analyzed using power law to consider the charge storage mechanism in the TiO_2_ electrodes. Electrochemical impedance spectroscopy (EIS) was measured in the same three‐electrode setup after CV measurements at open circuit potential (OCP). Spectra were collected with an AC signal amplitude of 10 mV in frequency intervals from 100 mHz to 100 kHz. The Nyquist plot was analyzed using Nova 2 (MetrOhm) software by modeling the equivalent circuit and finding the electrical parameters at the electrode‐electrolyte interface. Chronopotentiometry was measured using a two‐electrode cell with a 2 mm Pt rod as a counter electrode and reference electrode (See Figure S2, Supporting Information). TiO_2_/carbon felt electrode was used as a working electrode. The electrodes were prepared with 1 cm^2^ of carbon felt (specific surface area of 50 m^2^ g^−1^) loaded with 7 mg cm^−2^ of TiO_2_. For water electrolysis, the geometric area was kept constant at 0.5 cm^2^ (10 x 5 mm) and the volume of the electrode was 0.15 cm^3^ (thickness = 3 mm). Cycling was performed using three different current densities: 100, 500, and 1000 mA g^−1^ with a cut‐off voltage of 1.5 and −2.05 V for 1000 cycles in ambient temperature.

### Gas Chromatography–Mass Spectrometry

The identification of volatile compounds was performed using the Gas Chromatograph–Mass Spectrometer (GCMS‐QP2020 NX system, Shimadzu, Kyoto, Japan). The GCMS incorporated a dielectric barrier discharge ionization detector (BID), where samples were ionized by excited helium with an irradiation energy of 17.7 eV. An RT‐Msieve 5A column (30 m length, 0.53 mm inner diameter, 50 µm film thickness; Restek, USA) was used for the separation of permanent gases, with helium (grade 6.0) as the carrier gas at a linear velocity of 216.2 cm s^−1^. The oven was programmed to initially hold at 35 °C for 2 min, followed by a ramp up from 35 to 100 °C at a rate of 10 °C min^−1^, with an additional holding time of 6.5 min, resulting in a total run time of 1439 min. The injector temperature was set to 150 °C, operating in a split mode. The ion source temperature was maintained at 200 °C, while the interface temperature was set to 190 °C. The identification of the compounds was verified by comparing experimental retention indices to the NIST20 spectral library.

### Gas Production Rate and Efficiency Measurements

To determine the amount of released gasses and efficiency, the cell consisting of a polytetrafluoroethylene (PTFE) lid and body filled with an aqueous solution of 0.5 m H_2_SO_4_ was used. Both electrodes and the capillary were immersed in the electrolyte. The connecting tube of the medical syringe was inserted into the cell, but its tip was above the level of the electrolyte. During the measurement, an external power supply was connected to the electrodes and before the potential is applied, the liquid level in the capillary is adjusted to the lowest mark with a medical syringe. When the potential was applied, gases in the cell were being produced at 10 mA current (100 mA g^−1^) and the pressure rose above the liquid level, which raised the level in the capillary.

## Conflict of Interest

The authors declare no conflict of interest.

## Supporting information

Supporting Information

## Data Availability

The data that support the findings of this study are available from the corresponding author upon reasonable request.
