# Supplementary material for: Efficient Decoupled Electrolytic Water Splitting in Acid through Pseudocapacitive TiO2
Source: Adv Sci (Weinh). 2024 May 14;11(28):2401261. doi: 10.1002/advs.202401261 (PMC11267372; doi:10.1002/advs.202401261)
Supplement: Supplementary file 1 — Supporting Information [file ADVS-11-2401261-s001.docx]

**Supplementary information**

**Efficient Decoupled Electrolytic Water Splitting in Acid through Pseudocapacitive TiO_2_**

*Mairis Iesalnieks^[a]^, Mārtiņš Vanags^[a]^, Linda Laima Alsiņa^[a]^, Raivis Eglītis^[a]^, Līga Grīnberga^[b]^, Peter C. Sherrell,^[c]*^ Andris Šutka^[a]*^*

^[a]^ Institute of Materials and Surface Engineering, Faculty of Natural Sciences and Technology, Riga Technical University, P. Valdena Street 3/7, LV-1048, Riga, Latvia

^[b]^ Institute of Solid State Physics, University of Latvia, LV-1063, Riga, Latvia

^[c]^ Applied Chemistry & Environmental Science, School of Science, RMIT University, 124 La Trobe St, 3000, Melbourne, Australia

[andris.sutka@rtu.lv](mailto:andris.sutka@rtu.lv)

[peter.sherrell@rmit.edu.au](mailto:peter.sherrell@rmit.edu.au)


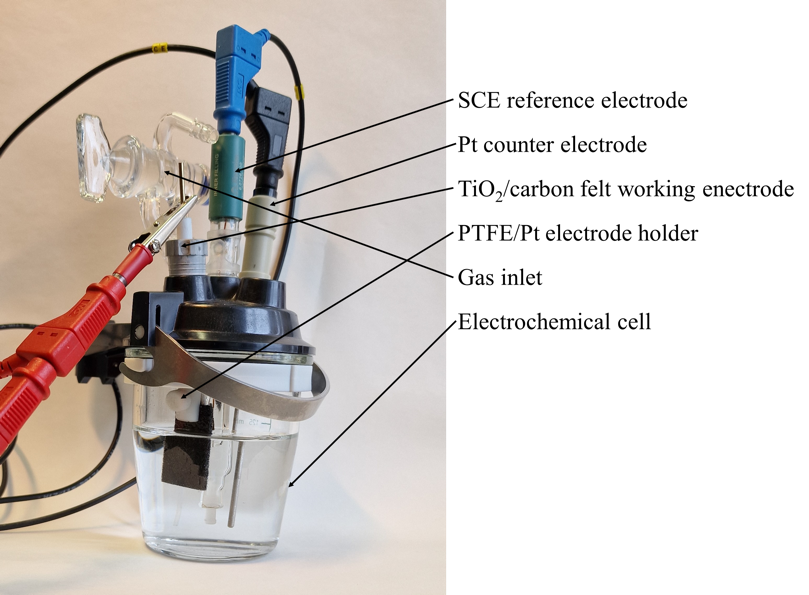


**Fig. S1**. Representation of the electrochemical set-up in three electrode system as used for cyclic voltammetry and electrochemical impedance spectroscopy.


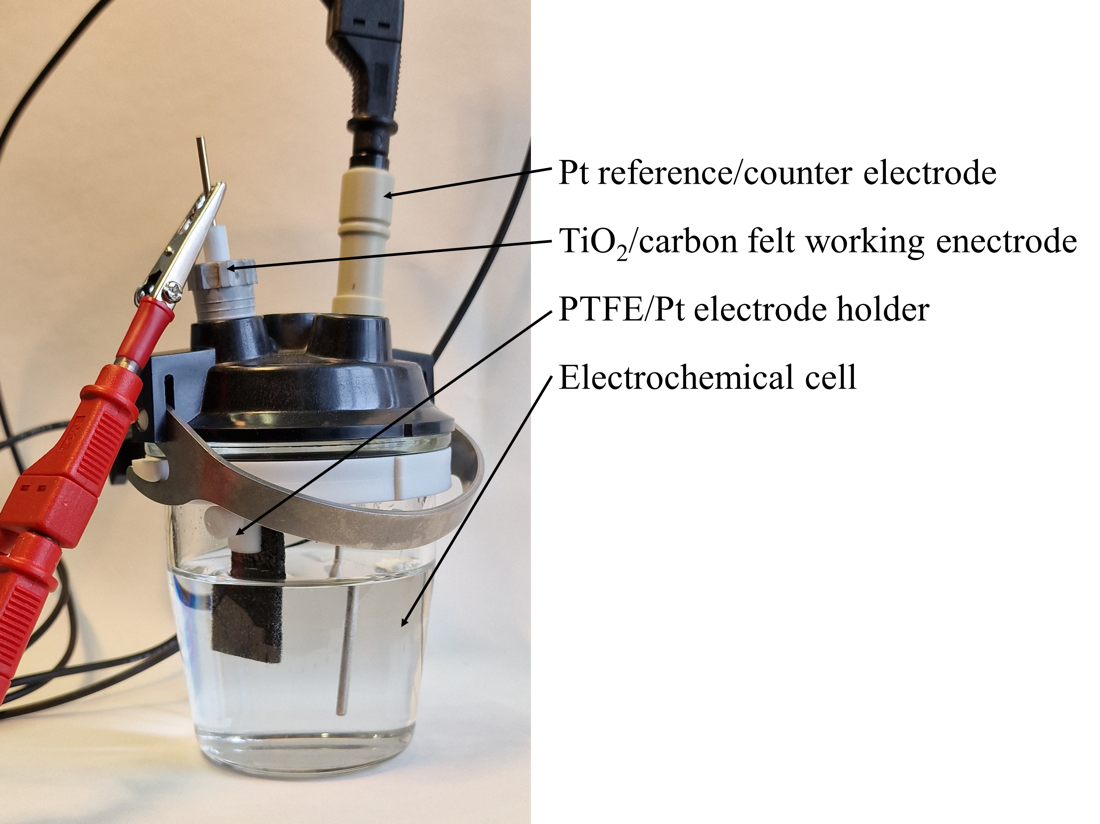


**Fig. S2**. Electrochemical cell set-up at two electrode system for chronopotentiometric measurements.


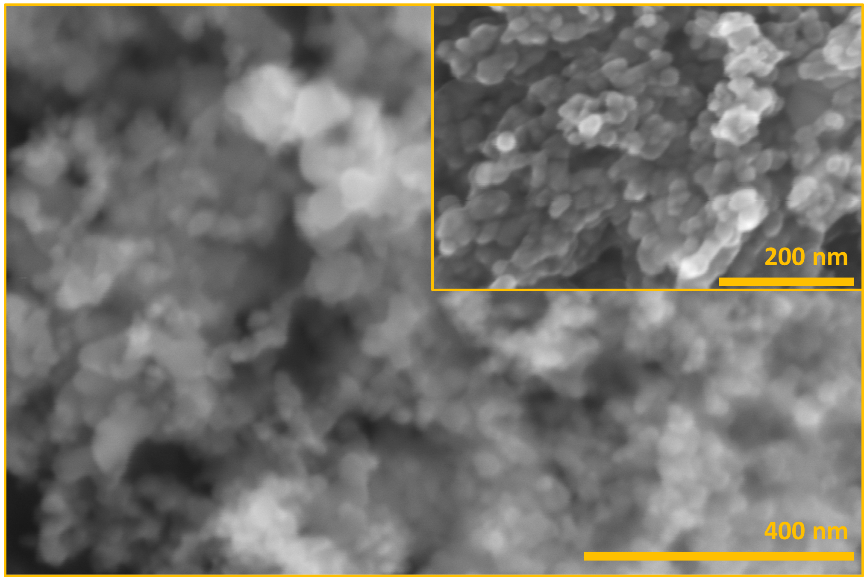


**Fig. S3**. SEM images of commercially available TiO_2_ P25 nanoparticles


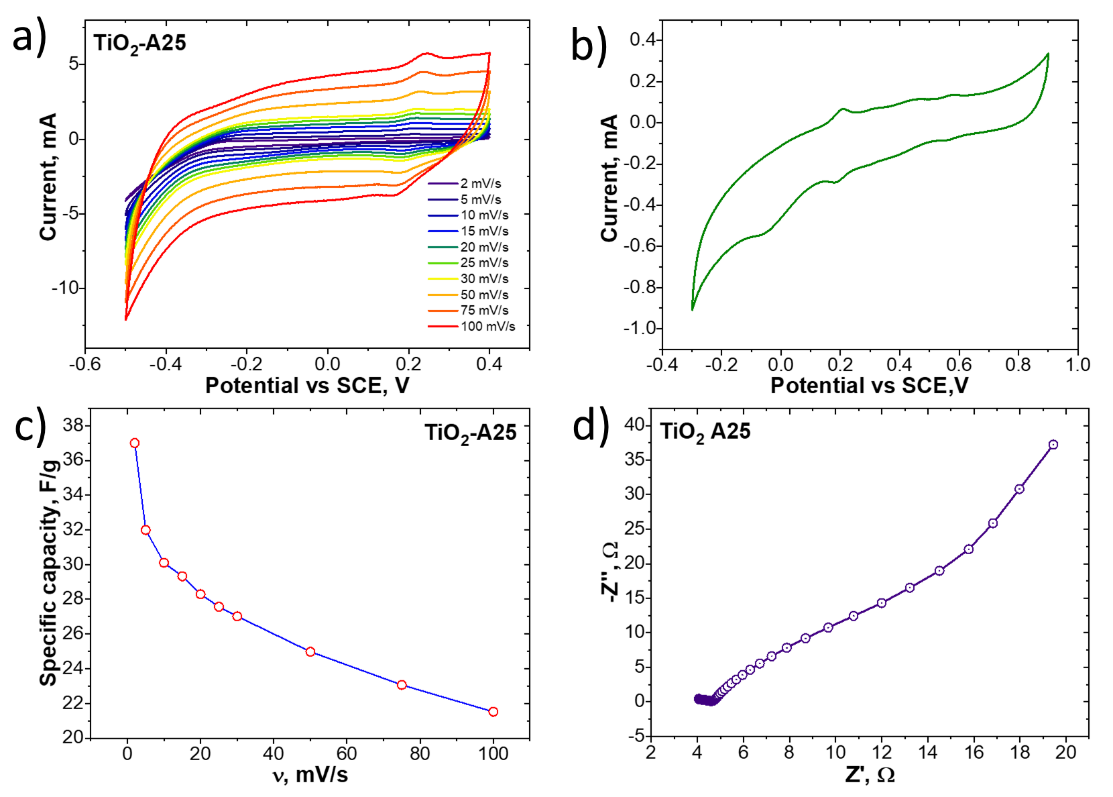


**Fig. S4**. Characterisation of commercially available pure anatase TiO_2_ nanoparticles using a) CV, b) CV scan at 5 mV/s, c) specific capacity dependency on scan rate, d) and EIS.

CV curves were taken at different scan rates to characterise the capacity of the TiO_2_ auxiliary electrode. CV curves are shown for all samples in Fig. S5. In the TiO_2_ P25 sample (Fig. S5 a), the CV curve forms an almost rectangular shape, indicating the nature of the electrical double-layer capacitance. As discussed above, TiO_2_ super-small nanoparticles reflect distinct redox processes related to the intercalation and deintercalation of H^+^ ions in the TiO_2_ material (Fig. S5 b, c).


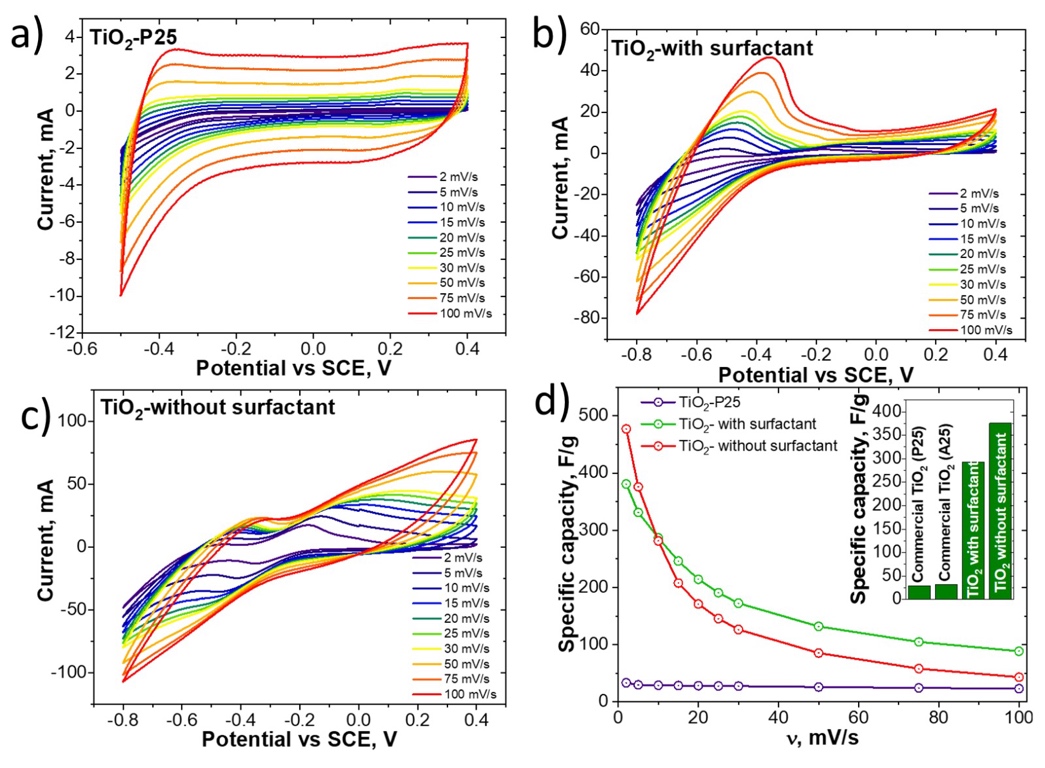


**Fig. S5**. CV at different scan rates for samples a) TiO2 P25, b) TiO2 with surfactant and c) TiO2 without surfactant. In d), capacitance decrement vs scan rate is shown. The inset figure shows the capacity for a particular sample at a 5 mV/s scan rate.

The specific capacitance was calculated from the CV curves (Figure S5 a-c) using the equation^1,2^:

$C= \frac{\int i\left( V \right)dV}{m\cdot\vartheta\cdot\Delta V}$ (3)

where the integral in the numerator of the equation indicates the total area under the CV curve, *ΔV* is the potential window (V), *ϑ* is the scan rate (V/s) and *m* is the mass of the active substance (g).

At 5 mV/s scan rate, the capacitance for the TiO_2_ without surfactant sample reaches 375 F/g, while for the TiO_2_ with surfactant sample, the capacitance at this scan rate is 326 F/g and TiO_2_ P25 only 30 F/g. In addition, the capacitance of an electrode fabricated from a commercially available anatase phase of TiO_2_ was tested to ensure that the pure anatase phase was not the main reason for the high capacitance. It can be seen in Fig. S5 d that a commercially available pure anatase phase has approximately the same capacitance as the P25 sample, confirming that the high capacitance for super-small nanoparticles is due to the microstructure, not the phase.


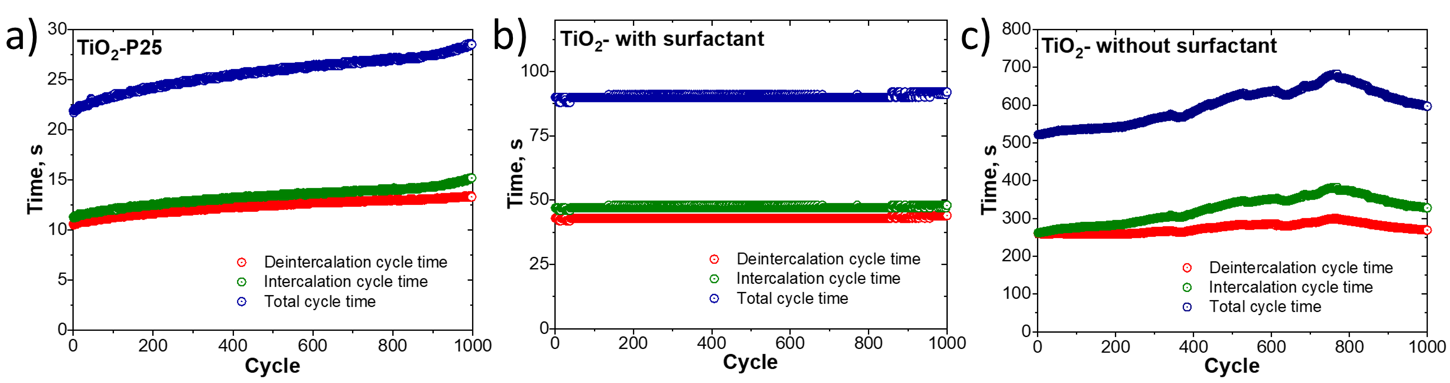


**Fig. S6**. The kinetics of cycle time changes for a) P25, b) TiO_2_ with surfactant and c) TiO_2_ without surfactant.


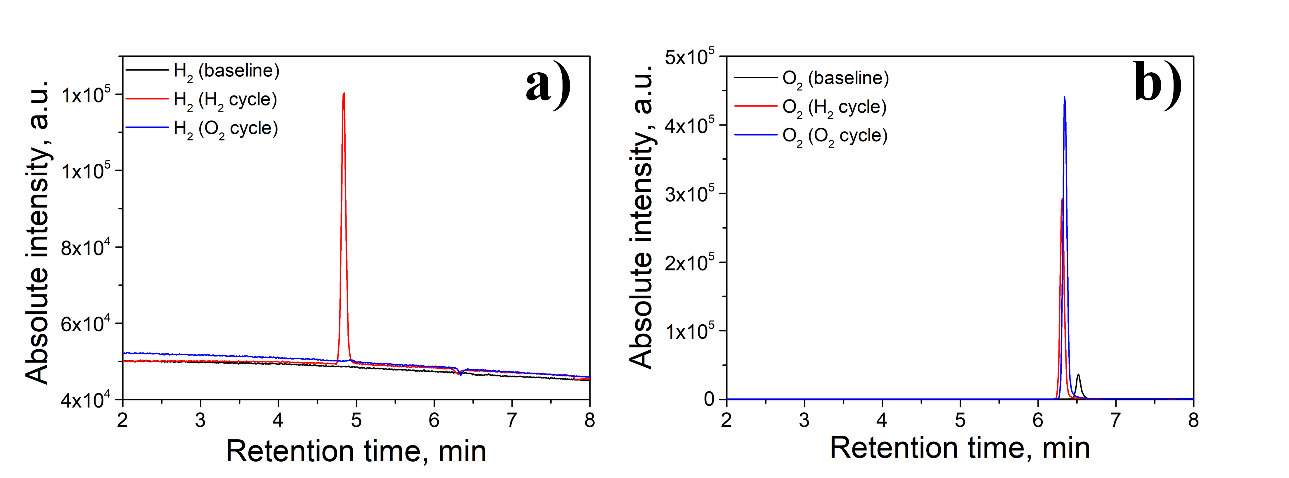


**Fig. S7**. Gas chromatography data a) hydrogen line and b) oxygen line.


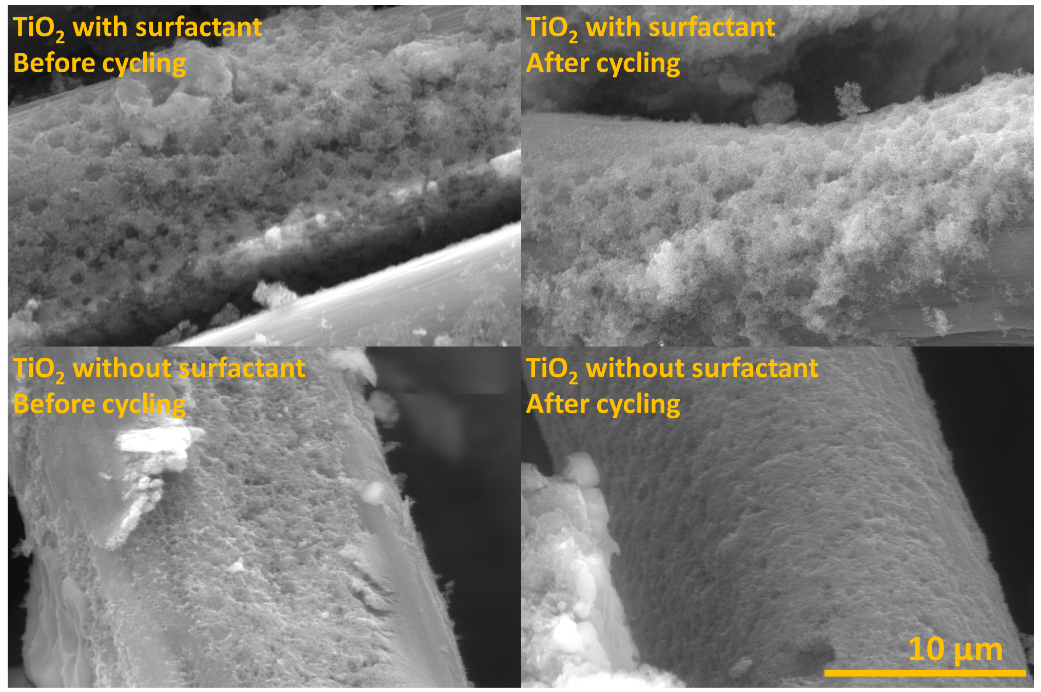


**Fig. S8**. SEM images of auxiliary electrodes before and after all cycles.

**Table S1**. Time to displace a volume of H_2_ gases and calculation of the faradaic efficiency

| Stripped TiO_2_ | | Non-stripped TiO_2_ | |
| --- | --- | --- | --- |
| Time, to produce 0.13 ml of H_2_, s | Faradaic efficiency, % | Time, to produce 0.13 ml of H_2_, s | Faradaic efficiency, % |
| 114 | 98.4 | 126 | 88.7 |
| 126 | 88.5 | 111 | 101.4 |
| 123 | 91.3 | 126 | 88.7 |
| 120 | 93.1 | 126 | 88.7 |
| 120 | 93.2 | 126 | 88.7 |
| 119 | 94.1 | 126 | 88.7 |
| 115 | 97.5 | 126 | 88.7 |
| 122 | 92.0 | 126 | 88,7 |
| 123 | 91.2 | 126 | 88.7 |
| 120 | 93.3 | 126 | 88.7 |
| 118 | 95.0 | 111 | 101.4 |


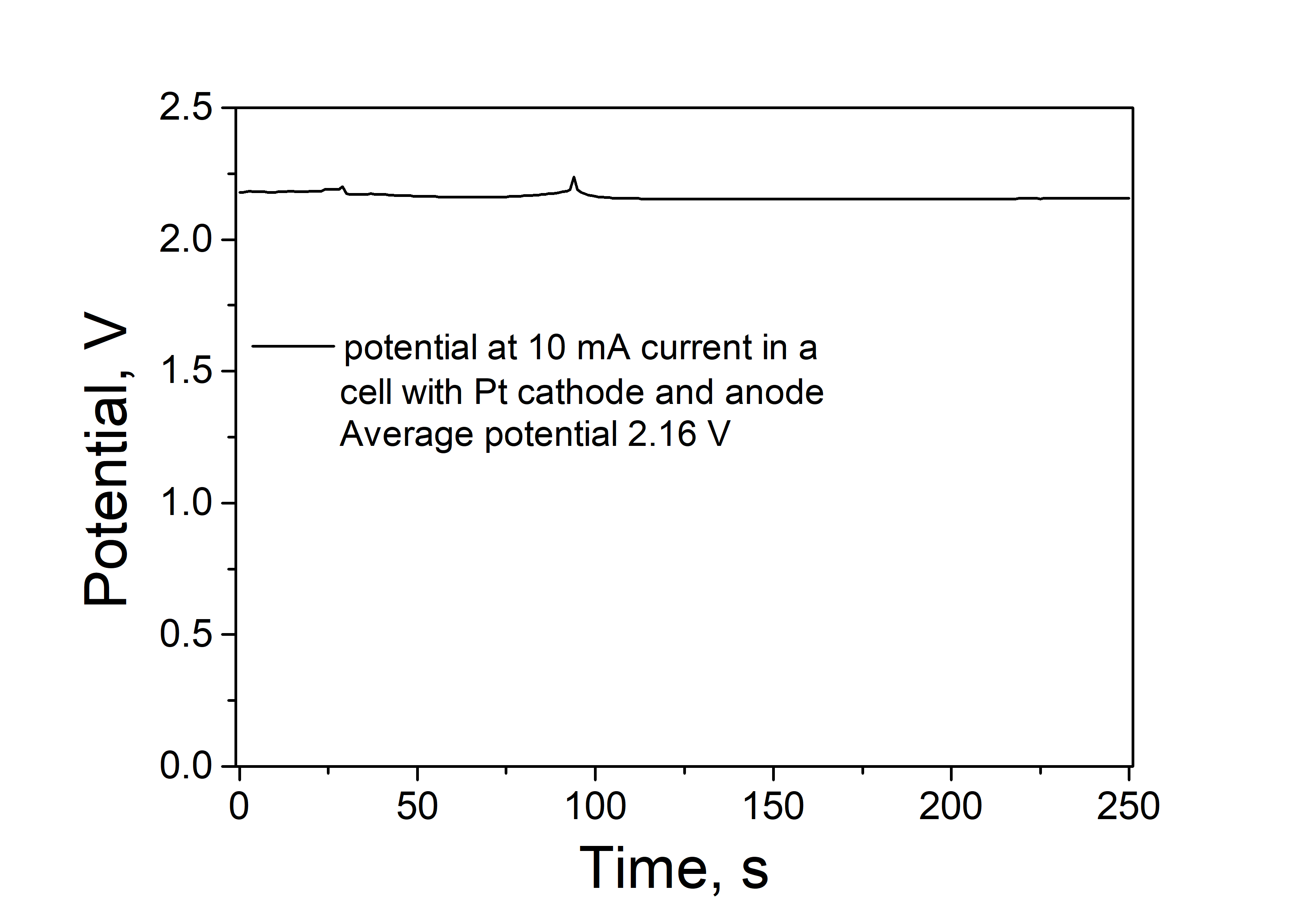


**Fig. S9**. Chronopotentiometry measurement of an electrochemical cell consisting of a Pt anode and cathode in an acidic medium.

**References**

(1) Lokhande, V.; Lokhande, A.; Namkoong, G.; Kim, J. H.; Ji, T. Charge Storage in WO3 Polymorphs and Their Application as Supercapacitor Electrode Material. Results Phys 2019, 12, 2012–2020. https://doi.org/10.1016/j.rinp.2019.02.012.

(2) Das, A. K.; Karan, S. K.; Khatua, B. B. High Energy Density Ternary Composite Electrode Material Based on Polyaniline (PANI), Molybdenum Trioxide (MoO3) and Graphene Nanoplatelets (GNP) Prepared by Sono-Chemical Method and Their Synergistic Contributions in Superior Supercapacitive Performance. Electrochim Acta 2015, 180, 1–15. https://doi.org/10.1016/j.electacta.2015.08.029.
